# Microscopic and molecular diagnoses of *Giardia duodenalis* in pet animals in Babylon Province, Iraq

**DOI:** 10.14202/vetworld.2023.2263-2270

**Published:** 2023-11-12

**Authors:** Shurook R. Idan, Mohammad H. Al-Hasnawy

**Affiliations:** 1 Department of Public Health, Babil Health Directorate, Ministry of Health, Iraq; 2 Department of Parasitology, College of Veterinary Medicine, Al-Qasim Green University, Iraq

**Keywords:** cat, dog, *Giardia duodenalis*, microscopic, molecular diagnosis

## Abstract

**Background and Aim:**

The breeding of pet animals, especially dogs and cats, in Iraq has increased recently. However, no epidemiological or molecular data exist regarding *Giardia duodenalis* infection in pet animals, particularly in Babylon Province. Therefore, this study aimed to detect *G. duodenalis* and its genotypes in pets using microscopic and molecular techniques.

**Materials and Methods:**

For microscopic examination, 150 pet fecal samples (dogs = 75, cats = 75) were examined from October 1, 2022, to March 30, 2023. Fourteen isolates of *G. duodenalis* (7 per host) were genetically characterized using *SSUrDNA* *gene* sequencing.

**Results:**

Microscopic examination revealed that the rates of *G. duodenalis* infection was 14.67% (11/75) and 12% (9/75) in dogs and cats, respectively. According to sex, the infection rate in dogs was 22.22% (8/36) for males and 7.69% (3/39) for females. Conversely, the infection rate in cats was 5.56% (2/36) for males and 17.95% (7/39) for females. The highest infection rates were recorded for animals under 6 months of age, with 16.67% (3/18) for dogs and 27.27% (3/11) for cats. In rural areas, the infection rate was higher than that in urban areas, with 17.65% (6/34) in dogs and 13.89% (5/36) in cats. For the molecular diagnosis, 14 isolates of *G. duodenalis* (7 per host) were genetically characterized using *SSUrDNA* gene sequencing. In dogs, the findings revealed specific genotypes, with D at 3/7 (42.86%) and C at 2/7 (28.57%). In addition, zoonotic genotype A was found in 2/7 (28.57%) of dogs. In cats, the specific assemblage F was present in 4/7 (57.14%), while zoonotic genotype A was found in 3/7 (42.86%).

**Conclusion:**

This study is considered the first in Babylon Province to detect *G. duodenalis* genotypes in pet animals (dogs and cats), as some have zoonotic genotypes that could transmit infections to humans. The results of this study illustrate the epidemiological importance of this parasite in this region.

## Introduction

*Giardia duodenalis* is a common protozoan that infects domestic and wild animals [1]. Animals with symptomatic or asymptomatic giardiasis pose a risk of infection to other humans and animals, and reinfection is common unless infective cysts are eliminated from the environment [2].

According to genetic research on certain genetic markers, *G. duodenalis* is classified as a species complex comprising eight genetic assemblages (A–H). Assemblages A and B are known to be harmful to humans and have zoonotic potential, as they have been found in animals and humans [3]. Other assemblages, such as C and D, are prevalent in dogs, while assemblage E infects domestic ruminants and pigs. Assemblage F infects cats, while assemblages G and H infect rats and marine mammals, respectively [4, 5].

Giardiasis can be diagnosed in the laboratory using fecal microscopic examination, various immunological-based tests, and molecular approaches. Polymerase chain reaction (PCR) is more accurate and useful for diagnosing *G. duodenalis* infections than enzyme-linked immunosorbent assay and fecal microscopy [6]. *Giardia duodenalis* is significant to public health and has economic implications for the country due to the threat of an outbreak and associated economic losses. However, there are no previous epidemiological or molecular data regarding *G. duodenalis* infection in pet animals in Babylon Province.

Therefore, this study aimed to detect and identify *G. duodenalis* genotypes in pets in this region using microscopic and molecular techniques.

## Materials and Methods

### Ethical approval

This study was approved by the Ethics Committee of the College of Veterinary Medicine/Al-Qasim Green University (No: 2022, October 11 2022).

### Study period and location

This study was conducted in Babylon Province from October 1, 2022, to March 30, 2023. For microscopic examination, 150 pet fecal samples (dogs = 75 and cats = 75) were collected in sterile plastic cups (approximately 10 g) to detect trophozoites and/or cysts of *G. duodenalis* in different locations (zoos, veterinary clinics, and houses). During collection, color and consistency were recorded for each host.

### Direct wet smear

A small amount of feces was collected using a pin stick, thoroughly mixed on a glass slide, and covered with a coverslip. The sample was examined under a microscope at 40× and 100× magnifications. Smears stained with Lugol’s iodine were prepared by mixing a small amount of feces with a drop of Lugol’s iodine stain on a glass slide, followed by microscopic examinations at 40× and 100× magnifications [7, 8].

### Concentration methods

#### Flotation technique

Fecal flotation based on zinc sulfate was conducted using 2–4 g of feces. The feces and zinc sulfate solution (specific gravity = 1.18) were centrifuged with a coverslip in place for 5 min at 200× *g*. After centrifugation, the coverslip was transferred to a glass slide and scanned for *G. duodenalis* trophozoites and/or cysts using a light microscope (Olympus/Japan) at 40× and 100× magnifications [9].

### Cyst measurement

Cysts were measured using a Leica microscope equipped with a digital camera (ScopeImage 9.0, China), which was accompanied by image processing software. The camera software was calibrated for all microscope lenses using a 0.01 mm stage micrometer (ESM-11/Japan).

### Molecular study

#### DNA extraction

Following the manufacturer’s instructions, DNA was extracted directly from 60 fecal samples (30 from dogs and cats each), including microscopically positive samples, for confirmation using (EasyPure^®^Stool Genomic DNA Kit, Transgen company, China).

### Conventional PCR

Conventional PCR was peformed to detect and identify *G. duodenalis* DNA using the primers RH11 and RH4 and the Giar primer of the *SSUrDNA* gene, following the protocol described by Hopkins. The primers used were RH11(F) 5′-CATCCGGTCGATCCTGCC-3′, RH4(R) 5′-AGTCGAACCCTGATTCTCCGCCCAGG-3′, and *Giar*(F) 5′- GACGCTCTCCCCAAGGAC -3′ (R)′5′- CTGCGTCACGCTGCTCG-3′ [10]. A 130 bp region of the PCR primer Giar [11] was utilized for the dog samples. For cat samples, we used the primary oligo RH11 as the secondary forward primer and GiarR as the secondary reverse primer, allowing for a longer secondary fragment at the 5′ end of the sequence, approximately 197 bp [12]. The PCR process involved an initial denaturation step at 96°C for 3 min, followed by 35 cycles of denaturation for 45 s at 96°C, annealing for 30 s at 50°C, and extension for 45 s at 72°C. The final extension step was conducted at 72°C for 7 min.

### DNA sequencing

DNA sequencing was conducted to identify *G. duodenalis*, and seven PCR-positive local isolate products were sent to Macrogene Company in Korea to identify *G. duodenalis* genotypes. A homology search was conducted using the Basic Local Alignment Search Tool available at the National Center for Biotechnology Information (NCBI, http://www.ncbi.nlm.nih.gov) and the BioEdit program, version 7.2. The results were compared with data obtained from the GenBank and the ExPASY program (SIB Swiss Institute of Bioinformatics, Switzerland) available on the NCBI website [13, 14].

### Phylogenetic tree

To compute evolutionary distances, a phylogenetic tree was constructed using the Unweighted Pair Group Method with Arithmetic Mean (UPGMA) method [15].

### Statistical analysis

Statistical analysis was conducted using the statistical package for the social science version 27 for Windows (IBM Corp., NY, USA) and Microsoft Excel 2010 (Microsoft Corp., Washington, USA) [16].

## Results

### Microscopic examination

**Figure-1:**
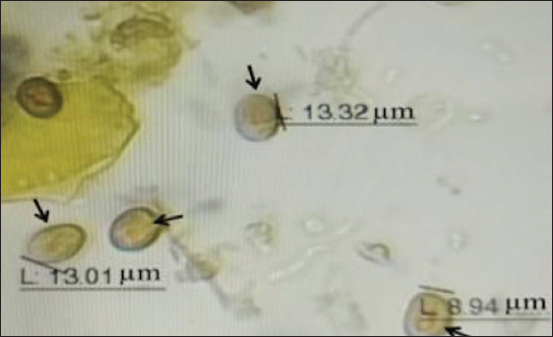
*Giardia duodenalis* cysts (as shown in black arrows) isolated from cat fecal samples by direct smear method with Iodine stain: 40×.

**Figure-2:**
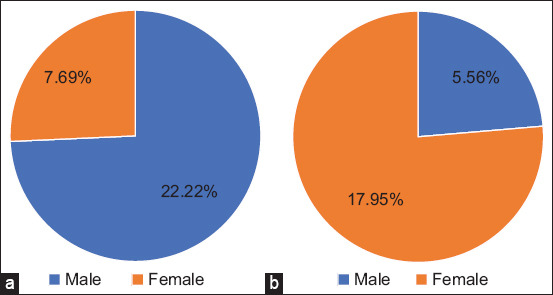
Infection rates of *Giardia duodenalis* according to sex with no significant differences (p ≤ 0.05). (a) In dogs (p = 0.076, X^2^ = 3.158). (b) In cats (p = 0.099, X^2^ = 2.723).

**Figure-3:**
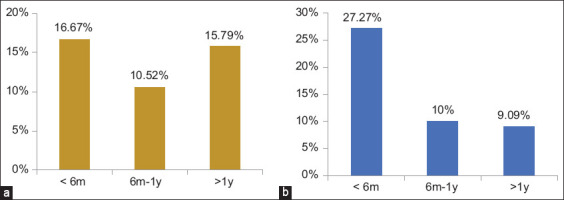
Infection rates of *Giardia duodenalis* according to age groups with no significant differences (p ≤ 0.05). (a) In dogs (p = 0.834, X^2^ = 0.411). B: In cats (p = 0.307, X^2^ = 2.73).

**Figure-4:**
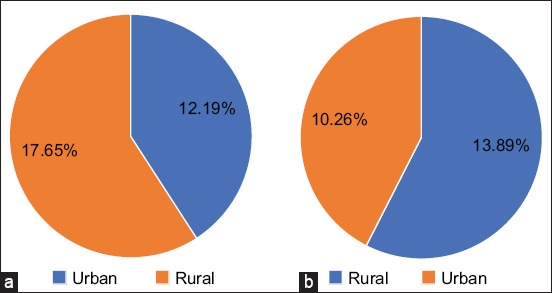
Infection rates of *Giardia duodenalis* according to area with no significant differences (p ≤ 0.05). (a) In dogs (p = 0.532, X^2^ = 0.441). (b) In cats (p = 0.629, X^2^= 0.234).

**Figure-5:**
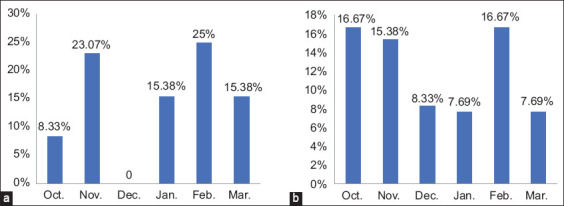
Infection rates of *Giardia duodenalis* according to months with no significant differences (p ≤ 0.05). (a) In dogs (p = 0.539, X^2^ = 4313). (b) In cats (p = 0.942, X^2^ = 1.63).

The microscopic examination results demonstrated that *G. duodenalis* cysts have an oval shape with four nuclei, an axostyl in the central cyst, and a thick wall that appears brown when stained with iodine. The average size of these cysts was 12.5 × 8.5 ± 1.5 mm (Figure-1). The rates of *G. duodenalis* infection were 14.67% (11/75) in dogs and 12% (9/75) in cats. According to sex, the infection rate in dogs was 8/36 (22.22%) for males and 3/39 (7.69%) for females. In cats, the infection rate was 2/36 (5.56%) for males and 7/39 (17.95%) for females, with no statistically significant differences (p ≤ 0.05) (Figure-2). The highest infection rates were observed in animals under 6 months of age, with 3/18 (16.67%) for dogs and 3/11 (27.27%) for cats. No statistically significant differences were observed (p ≤ 0.05) (Figure-3). Rural areas had a higher rate of infection than urban areas, with 6/34 (17.65%) in dogs and 5/36 (13.89%) in cats, and no statistically significant differences were observed (p ≤ 0.05) (Figure-4). The highest infection rate in dogs was observed in February (3/12, 25%). Similarly, the highest infection rate in cats was observed in February and October (2/12, 16.67%), with no statistically significant differences (p ≤ 0.05) (Figure-5).

### Molecular diagnosis

**Figure-6:**
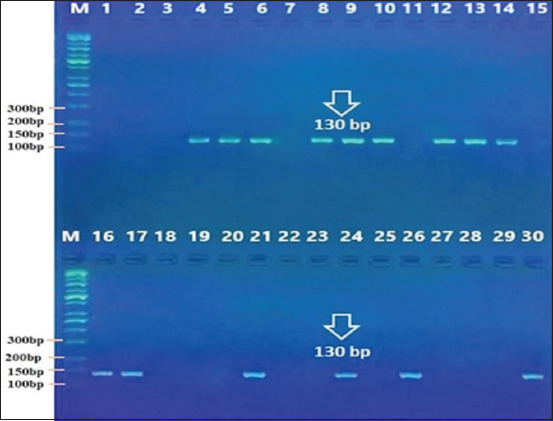
Polymerase chain reaction product of *Giardia duodenalis* in dogs, the band size (130) bp. The product was electrophoresed on 1.5 % agarose. TBE 1× buffer for 1:30 h. M: DNA ladder (100).

**Figure-7:**
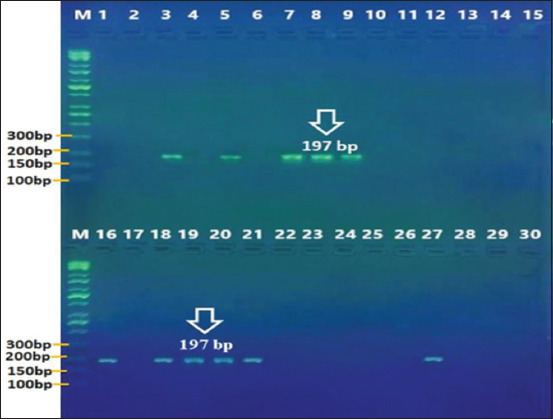
Polymerase chain reaction product the band size (197 bp) (cats). The product was electrophoresed on 1.5 % agarose. TBE 1× buffer for 1:30 h. M: DNA ladder (100).

The molecular diagnosis results showed that the PCR amplification of the *SSUrDNA* gene was positive for 50% (15/30) of dogs and 37% (11/30) of cats (Figures-6 and 7).

### Sequence analysis of *G. duodenalis*

**Figure-8:**
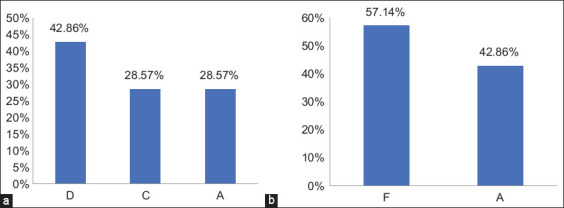
Genotypes of *Giardia duodenalis* in pet animals, (a) in dogs, (b) in cats.

To investigate *G. duodenalis* genotypes in pet animals in Babylon Province, 14 isolates of *G. duodenalis* (7 per host) were genetically characterized using *SSUrDNA* *gene* sequencing. In dogs, sequencing revealed specific genotypes D (3/7, 42.86%) and C (2/7, 28.57%), along with zoonotic genotype A (2/7, 28.57%) of *G. duodenalis*. In cats, the specific assemblage F was present in 4/7 (57.14%), while zoonotic genotype A was found in 3/7 (42.86%) (Figure-8).

*Giardia duodenalis* isolates were submitted to the NCBI GenBank database, and GenBank accession numbers were obtained for 14 *G. duodinalis* isolates: OR072983.1, OR072984.1, OR072985.1, OR072986.1, OR072987.1, OR072988.1, and OR072989.1 for dogs, and OR072997.1, OR072998.1, OR072999.1, OR073000.1, OR073001.1, OR073002.1, and OR073003.1 for cats. Sequences of the isolates were aligned with reference isolates of *G. duodenalis* that had previously been recorded in GenBank.

### Phylogenetic tree

**Figure-9:**
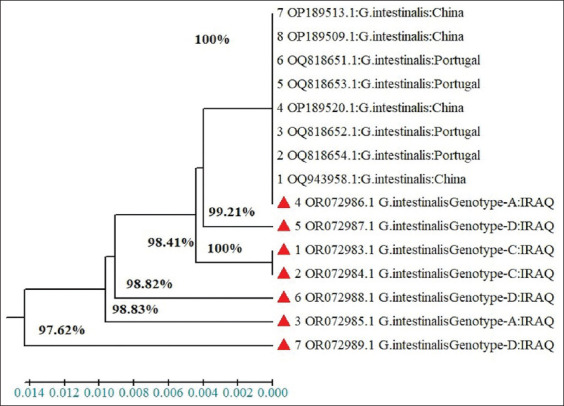
Phylogenetic tree analysis based on the partial sequence of *SSUrDNA* gene explains identity between the local isolates of *Giardia duodenalis* in dogs and NCBI-BLAST isolates.

**Figure-10:**
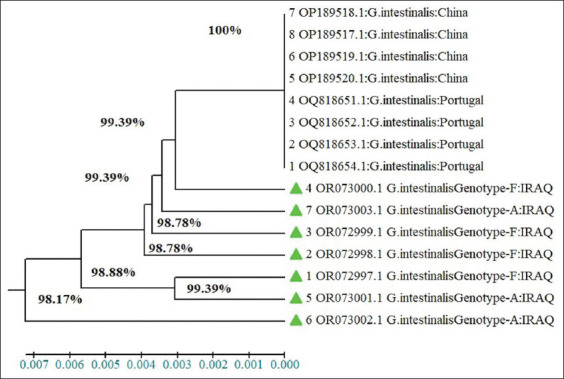
Phylogenetic tree analysis based on the partial sequence of *SSUrDNA* gene explains identity between the local isolates of *Giardia duodenalis* in cats and NCBI-BLAST isolates.

The evolutionary history was inferred using the UPGMA method. The optimal tree with the sum of branch length = 0.02117480 is shown. According to the phylogenetic tree, the *G. duodenalis* isolates were shown to be closely related to strains worldwide (97.6%–100% and 98%–100% for dogs and cats, respectively) (Figures-9 and 10).

## Discussion

This study represents the first contribution to the knowledge of *G. duodenalis* genotypes in pet animals in Babylon Province. Correctly genotyping *G. duodenalis* isolates is crucial for providing accurate information about the risk of zoonotic transmission, host specificity, and the sources of infection during outbreaks. This analysis could also help to define the role of *G. duodenalis* as an animal pathogen that harms animal health.

Through microscopic examinations, the infection rate of *G. duodenalis* observed in dogs was 14.67%. This result was significantly aligned with a previous study by Hassan [17] that reported a 16% infection rate in Erbil, which was higher than the rate reported in another study conducted in Duhok Province (5.2%) [18]. However, the infection rate observed in this study was lower than that recorded in Basrah (75.55%) [19]. In the present study, the infection rate in cats was 12% which is highly consistent with the results of De Waal *et al*. [20] in Ireland but lower than the rate observed in another study in Wasit Province (50.4%) [21]. In Iraq, *G. duodenalis* is highly endemic due to unsanitary conditions and favorable climates, and its prevalence varies across studies depending on geographical areas, local animal habitats, animal populations, and seasonal variations throughout the year. Notably, the infection rate of *G. duodenalis* is higher in stray cats and dogs than in pets [22].

Infection rates were observed to be higher in male dogs than in females. This result was consistent with that of Hassan [17] in Erbil, where the infection rate was higher in male dogs (16%) than in female dogs (7%). In contrast, Jasim and Faraj [23] found that the infection rate was higher among female dogs than male dogs in Baghdad. Male dogs have been reported to be at a higher risk due to their increased activity over larger areas, making them more relevant pathogen carriers than females [22, 24]. Conversely, the infection rate in cats was observed to be higher in females than in males in this study, which is consistent with the results of the previous studies in Iraq [21], Mexico [25], and Turkey [26]. However, Veyna-Salazar and Cantó-Alarcón [27] found that sex did not show a significant correlation with the occurrence of *G. duodenalis* in Queretaro, with infection rates similar in male (24%) and female (25%) cats (p ≤ 0.05). The high rate of *G. duodenalis* infection in female cats was due to some hormones in the females triggering a decrease in the immune defense mechanism [21]. This study demonstrates that the highest infection rates in pets were observed in the age group under 6 months. These findings agree with the previous studies conducted in Iraqi cities by Naser and Wadood [19], Alhayali *et al*. [22], Jasim and Faraj [23], and Latif *et al*. [28]. *Giardia duodenalis* infections in dogs and cats have been investigated worldwide, with the infection prevalent in younger animals [26, 29–31]. The higher infection rate in young animals than in older animals can be attributed to the varying immune status of the animals [32, 33].

For pets, the highest infection rates were recorded in rural areas. These findings align with a study by Al-Sherefy and Al-Hamairy [34] in Babylon Province, where the infection rate in rural areas was higher than that in urban areas. Similar trends have been observed in studies involving humans, dogs, and cats [35–38]. The high infection rates in rural areas may be due to several factors, including a lack of clean drinking water, dependence on river water as a direct water source, contact with contaminated soils in gardens and farms harboring parasite cysts, close interaction with animals that serve as reservoirs for the parasite, the use of animal waste as organic fertilizer, and low health and cultural levels in rural populations [35]. For dogs, the highest infection rate was observed in February (25%).

Similarly, the highest infection rates in cats occurred in February and October. These results are consistent with another study that confirmed a higher prevalence of Giardia infections during the rainy season [39]. Seasonal variations in infection rates may be influenced by changes in temperature, rainfall, and moisture, which can facilitate the spread of waterborne diseases. These seasonal variations could become significant in certain areas if climate change continues to cause a shortage of clean surface water [40].

Regarding the molecular results, PCR was used to amplify genes encoding *SSUrDNA*. The *SSUrDNA* locus is a highly sensitive screening tool for *G. duodenalis* obtained directly from feces and is the traditional gene sequence used for identification and phylogenetic analyses [41]. The conserved *SSUrDNA* gene is a common marker for assemblage differentiation (mostly genotyping) of *G. duodenalis* [3, 13]. In dogs, the highest percentage of isolates exhibited the dog-specific genotype assemblages C and D, followed by the potential zoonotic genotype A. Various studies in different geographical areas, including Poland and Italy, have also shown that the C and D genotypes were predominant [33, 42]. However, this study detected zoonotic genotype A in a lower percentage. A previous study by Hassan [17] in Erbil Governorate reported that the zoonotic genotype A was the most common type among stray dogs. Another study in China by Zheng *et al*. [43] found that most dogs in that region exhibited zoonotic A clustering, consistent with findings by Zhao *et al*. [16] in China. Among the genotype groups in dogs, the prevalence of genotype A was significantly higher compared to other groupings. This high variability in *G. duodenalis* genotypes may depend on factors related to kennel management, genotypes affecting newly arrived dogs, and the potential for these genotypes to spread to other dogs. The increased occurrence of zoonotic genotypes in dogs in recent years has been suggested to be due to more frequent cross-species transmission of *G. duodenalis* between humans and dogs [44]. In cats, the percentage of specific assemblage F was higher than that of zoonotic genotype A. This pattern is consistent with findings in the previous studies by Al Mosawy *et al*. [21], Piekara-Stępińska *et al*. [33], and Cai *et al*. [44], where assemblage F of *G. duodenalis* was mainly detected in cats, followed by assemblage A. Conversely, an examination of feline fecal samples from Greece revealed mostly genotype A and rare cases of assemblage F [45]. However, another study proved that genotypes A or B are common in pet animals, suggesting a potential zoonotic risk and implications for human health [46].

According to the phylogenetic tree, the *G. duodenalis* isolates in this study exhibited similarity to sequences deposited in GenBank, with the maximum homology of the *SSUrDNA* gene sequences between the Iraqi *G. duodenalis* strain and strains worldwide being 97.6%–100% for dogs and 98%–100% for cats. The phylogenetic analysis results confirmed minimal differences between the Iraqi strains of *G. duodenalis* and those from other countries. This genetic variation may be due to differences in the area size of the reference sequence and in the geographical areas where the isolates were collected. Various methods were used for the genetic analysis, including PCR-based gene sequencing of partial or complete genes. The results of this study align with studies that have reported the existence of genetic variation and phylogenetic relationships among *G. duodenalis* populations worldwide [21, 47, 48].

## Conclusion

These findings suggest that transmitting zoonotic genotypes from pet animals to humans could have epidemiological significance. This study also represents the first contribution to the knowledge of *G. duodenalis* genotypes in pet animals (dogs and cats) within Babylon Province.

## Authors’ Contributions

MHA: Conceptualized and designed the study and analyzed the results. SRI: Data collection, analysis, and interpretation, and drafted the manuscript. Both authors have read, reviewed, and approved the final manuscript.

## Data Availability

The datasets used and/or analyzed during the current study are available from the corresponding author on reasonable request.
